# Marine Bioluminescence: Simulation of Dynamics within a Pump-Through Bathyphotometer

**DOI:** 10.3390/s24061958

**Published:** 2024-03-19

**Authors:** Austin Thombs, Igor Shulman, Silvia Matt

**Affiliations:** 1 Naval Research Laboratory, Stennis Space Center, MS 39529, USA; silvia.matt@nrlssc.navy.mil; 2 Department of Aerospace Engineering, Texas A&M University, College Station, TX 77840, USA

**Keywords:** bioluminescence, bathyphotometer, computational fluid dynamics, rate of strain, residence time

## Abstract

Bioluminescence is light produced by organisms through chemical reactions. In most cases, bioluminescent organisms produce light in response to mechanical stimulation, including from shear around objects moving in the water. Many phytoplankton and zooplankton are capable of producing bioluminescence, which is commonly measured as bioluminescence potential, defined as mechanically stimulated light measured inside of a chambered pump-through bathyphotometer. We have developed a numerical model of a pump-through bathyphotometer and simulated flow using Lagrangian particles as an approximation for bioluminescent marine plankton taxa. The results indicate that all particles remain in the detection chamber for a residence time of at least 0.25 s. This suggests that the total first flash of bioluminescent autotrophic and heterotrophic dinoflagellates will be measured based on the existing literature regarding their flash duration. We have found low sensitivity of particle residence time to variations in particle size, density, or measurement depth. In addition, the results show that a high percentage of organisms may experience stimulation well before the detection chamber, or even multiple stimulations within the detection chamber. The results of this work serve to inform the processing of current bioluminescent potential data and assist in the development of future instruments.

## 1. Introduction

Bioluminescence (BL) is light produced by organisms through chemical reactions in response to mechanical, chemical, and optical changes within their environment, as well as an indicator for predator–prey interactions and mating [1,2]. In this work, we only consider bioluminescent organisms, specifically those that produce light in response to mechanical stimulation, including from shear around moving objects in the water. Many species of phytoplankton (primarily autotrophic and mixotrophic dinoflagellates) and zooplankton (including heterotrophic dinoflagellates, copepods, euphausiids, and many gelatinous organisms) are capable of producing mechanically stimulated bioluminescence, which is commonly measured as BL potential, defined as mechanically stimulated light measured inside of a chambered pump-through bathyphotometer. Most pump-through bathyphotometers pull ocean water into a closed chamber, where the marine organisms are mechanically stimulated to produce light upon entry into the chamber [3,4,5]. The stimulation is achieved either through a pump, rotating impellers, or through the introduction of grid-generated turbulence.

The Underwater Bioluminescence Assessment Tool (UBAT) is the only currently existing and commercially available pump-through bathyphotometer [6]. We note the importance of the bathyphotometer systems that preceded the UBAT, many of which have extensive data repositories and complementary experimental data [4]. However, none are commercially available, and most have not been in use for many years. In the UBAT, oceanic water is entrained into an S-shaped intake that is designed to prevent pre-stimulation of organisms as they travel to the detection chamber. The inlet also acts as a light baffle to minimize ambient light collected by the instrument. To enter the detection chamber, particles contained in the water sample pass through a high-speed impeller that produces mechanical stimulation. The UBAT collects data on BL potential in units of photons/s. The BL potential measured by the bathyphotometer thus represents the sum of light emitted by different organisms in the detection chamber. Usually, zooplankton emit bright flashes (larger than $10^{10}$ photons/s), while most dinoflagellate species emit flashes that produce less than $10^{9}$ photons/s. However, several factors have an effect on how well the BL potential recorded in pump-through bathyphotometers correlates to the total light output of a given organism.

There are several known challenges that affect the interpretation of data collected with pump-through bathyphotometers (including the UBAT), as listed below [7]:

(1) The intake of the bathyphotometer can be avoided by fast-swimming organisms.
(2) The residence time of the organisms in the detection chamber might be inappropriate. The Total Mechanically Stimulated Light (TMSL) of an organism is a measure of its bioluminescent capacity, defined by the number of flashes produced by the organism, the duration of the flash, and the maximum intensity of the flash [8]. If residence time is low, some of this TMSL may not be recorded in the detection chamber.
(3) Some organisms can be pre-stimulated prior to reaching the detection chamber; therefore, some light will not be recorded in the detection chamber.
(4) Large volumes of seawater should be sampled to obtain statistically significant estimates of BL potential, so bathyphotometers should pump through large volumes of water.

These challenges create uncertainties in understanding what fraction of an organism’s TMSL is actually measured by pump-through bathyphotometers. In the present paper, we address the following questions:

(1) What is the distribution of residence time for the organisms in the detection chamber of a pump-through bathyphotometer?
(2) What is the rate of strain distribution recorded at the inlet, and does it facilitate the possibility of pre-stimulation?
(3) What rate of strain do organisms experience in the detection chamber, and can it cause multiple stimulations for some organisms?

To address the above questions, we developed a numerical model of a pump-through bathyphotometer, using the UBAT as a reference. For the remainder of this paper, we refer to the numerical model of the bathyphotometer as the SIM-BATH. We conducted Computational Fluid Dynamics (CFD) simulations of flow through the SIM-BATH, using Lagrangian particles as an approximation for bioluminescent marine taxa. From these simulations, we estimated the distribution of residence times for organisms in the detection chamber of the SIM-BATH, and we provide a statistical analysis of the rate of strain experienced by particles passing through the inlet and the detection chamber. Furthermore, we assess the sensitivity of results to changes in the density and diameter of particles, as well as to the instrument depth during deployment.

## 2. Materials and Methods

### 2.1. SIM-BATH Geometry

**Figure 1:**
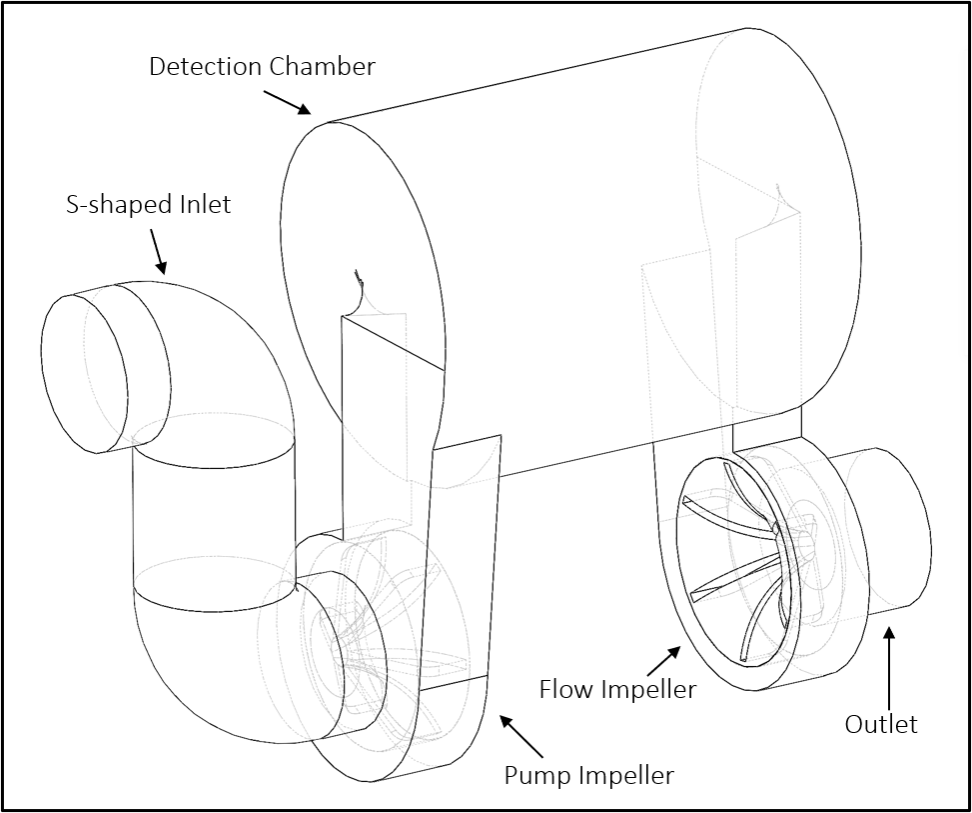
View of the SIM-BATH model domain, including hidden faces.

The UBAT bathyphotometer has two high-speed rotating impellers: a pump impeller to mechanically stimulate marine organisms and a flow impeller to maintain a specific flow rate through the instrument. Oceanic water enters the UBAT through the inlet into the S-shaped baffle that ends at the first impeller. The first impeller, called the pump impeller, spins at 1200 rpm and forces fluid into the detection chamber with a volume of 440 cm^3^. The flow impeller rotates at 600 rpm and redirects particles through the outlet. Measurements of the UBAT’s S-shaped inlet, impellers, and detection chamber were used to create the CAD geometry for a numerical model approximating the UBAT, which we refer to as the SIM-BATH. The SIM-BATH has all elements of a pump-through bathyphotometer, including an S-shaped inlet, two pumps for stimulation and flow control, a detection chamber, and an outlet. The resulting geometry, which comprises the internal fluid domain of the SIM-BATH, is shown in Figure 1.

### 2.2. Computational Methods

We used a finite-volume Navier–Stokes solver (STAR CCM 2021.2) for the modeling of fluid flow inside the SIM-BATH. The numerical model solves the unsteady Navier–Stokes equations given in Equations (1) and (2), using the finite volume method (FVM) with an implicit scheme. Here, ρ denotes density, $\vec{v}$ represents the fluid velocity vector, σ is the symmetric stress tensor, and $\vec{F_{b}}$ denotes body force. Flow is considered incompressible. Turbulence in the SIM-BATH is modeled with a Reynolds-Averaged Navier–Stokes (RANS) approach and the κ-ω SST model [9]. We used structured hexahedral cells to improve orthogonality in the volume mesh. The specifics of the turbulence model, grid design, and residuals are presented in Appendix A.1, Appendix A.2, Appendix A.3 and Appendix A.4.

(1) $$\frac{\partial \rho }{\partial t}+\nabla \cdot \left(\rho \vec{v}\right)=0$$

(2) $$\frac{\partial \rho \vec{v}}{\partial t}+\vec{v}\left(\nabla \cdot \rho \vec{v}\right)=\nabla \cdot \sigma +\vec{F_{b}}$$

The FVM solution of the flow field is subject to boundary conditions and initial conditions. At the inlet and outlet, the boundary is defined with a pressure condition. The pressure is specified and kept the same on both boundaries, and all other properties are extrapolated from interior cells. All other boundaries—those delineating the SIM-BATH surface—are defined as no-slip walls. This selection of boundary conditions means that the volumetric flow rate is not explicitly defined, and it is instead allowed to adjust freely based on flow impeller motion. The model is validated by evaluating the convergence of the volumetric flow rate as a function of grid size. The initial condition for the fluid velocity field is $\vec{v_{f}}=0$ throughout the SIM-BATH.

The dynamics of the bioluminescent organisms throughout the detection chamber are modeled with a particle tracking routine. A Lagrangian multiphase model was used for particle tracking. In this model, the Lagrangian particles are unidirectionally coupled to the RANS simulation, meaning the flow dynamics drive the particle motion but not vice versa. The equation of motion for the Lagrangian particles is given in Equation (3):

(3) $$m_{p}\frac{d\vec{v_{p}}}{dt}=\vec{F_{D}}+\vec{F_{P}}+\vec{F_{G}}$$

where $\vec{F_{D}}$ and $\vec{F_{P}}$ are surface force vectors corresponding to the effects of drag and pressure, $\vec{F_{G}}$ is a body force vector representing the force of gravity, $m_{p}$ is the mass of the particle, and $\frac{d\vec{v_{p}}}{dt}$ is the time rate of change of the particle’s velocity vector. The surface and body force vectors are described in more detail in Appendix A.5.

The sum of these forces at each time step is substituted into the equation of motion, from which an acceleration can be calculated. From the particle velocity calculated in Equation (5), we can extrapolate the particle displacement over the current time step. We assigned initial conditions to the Lagrangian particles at the time step corresponding to one second of model time (at which point the SIM-BATH had reached its operating flow rate) by seeding 1000 particles in a uniform distribution on the inlet boundary. Each particle was given an initial velocity in the direction of flow in the chamber as given by Equation (4) to account for acceleration prior to entering the SIM-BATH, where $\vec{A_{i}}$ is the inward-pointing area vector of the inlet and $\frac{dm}{dt}$ is the average mass flow rate.

(4) $$\vec{v_{p}}=\frac{1}{\rho \vec{A_{i}}}\frac{dm}{dt}$$

### 2.3. Residence Time

The residence time of a particle in the SIM-BATH is defined as the time during which that particle remains in the detection chamber. As a result, the distribution of the residence time for the ensemble of particles entering the SIM-BATH was estimated. The UBAT evolved from the Multipurpose Bioluminescence Bathyphotometer (MBBP) developed at UCSB [3]. An analytical equation for the percent of particles remaining in the detection chamber of the MBBP was proposed under the assumption of an already well-mixed detection chamber, where $n_{0}$ is the initial number of particles, *n* is the number of particles remaining at time *t* from the initial time, $\frac{1}{\rho }\frac{dm}{dt}$ is the volumetric flow rate, and *V* is the volume of the detection chamber. We compared the residence time distribution given in Equation (5), using the SIM-BATH flow rate and the volume of the detection chamber, with the corresponding distribution based on the CFD modeling.

(5) $$\frac{n}{n_{0}}=exp\left(-\frac{t}{\rho V}\frac{dm}{dt}\right)$$

### 2.4. Estimation of Rate of Strain

The rate of strain tensor, denoted as *L*, is expressed as the gradient of the velocity vector $\vec{v}$ in Equation (6):

(6) $$L=\nabla \vec{v}$$

The magnitude of the symmetric rate of strain tensor |E| is given as follows:

(7) $$E=\frac{1}{2}\left(L+L^{T}\right)$$

(8) $$\left|E\right|=\sqrt{E:E}$$

**Figure 2:**
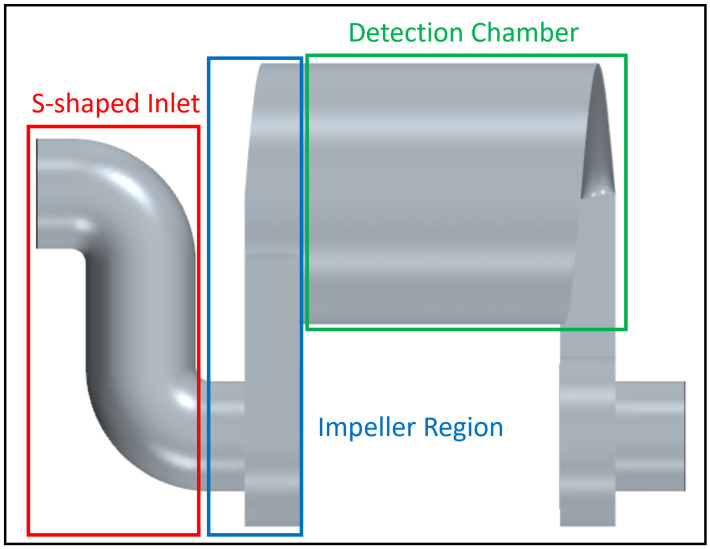
Defined regions for rate of strain analysis.

The rate of strain tensor *L* is recorded for each region as shown in Figure 2, from which |E| is derived. Regions of interest are the S-shaped inlet, the area around the pump impeller, and the detection chamber. The size of the impeller region is extended upwards and leftwards of the impeller itself to capture the rates of strain immediately before and after the impeller.

### 2.5. Design of Model Runs

As stated in the Introduction, the objective of this paper is to estimate the distributions of the following quantities: the residence time for the plankton in the detection chamber, the rate of strain experienced by plankton in the S-shaped inlet, and the rate of strain experienced by plankton in the detection chamber. Lagrangian particles were placed in the flow to track the trajectory of simulated organisms within the SIM-BATH. In oceanographic applications, Lagrangian particle tracing is a standard approach for simulating the dynamics of marine organisms in oceanic flow. The Lagrangian particle model does not include particle-to-particle or particle-to-fluid interactions. For this reason, the particle tracking method is unidirectional, and the path of a given particle will be the same if multiple sizes and densities are combined in the same run, or if multiple runs are used with uniform parameters for each run, as was performed in this work. Table 1 provides a summary of the model runs, which assess sensitivity to diameter, density, and depth of deployment (pressure).

For Run 1, the baseline run, the intention was to approximate the flow of massless particles through the SIM-BATH. For this reason, we used a small particle diameter $D_{p}$ = 2 μm with a density $\rho_{p}$ = 1000 kg/$m^{3}$ to mitigate buoyant forces. For a particle with these properties, the expected mass is on the order of $10^{-10}$ grams.

In coastal regions, the primary source of mechanically stimulated bioluminescence is dinoflagellates, which generally range in size from about 15 μm to 100 μm but can reach sizes approaching 1 mm [10]. With many small-volume bathyphotometers like the UBAT, large organisms are likely to avoid the inlet [7]. As a result, we considered particles with diameters closer to the mean. Runs 2 and 3 were replicas of Run 1 but with particle diameters defined as $D_{p}$ = 20 μm and $D_{p}$ = 200 μm, respectively. Comparisons of Runs 1–3 highlight the impact of particle size on the residence time and the rate of strain experienced while passing through the SIM-BATH.

The densities of phytoplankton depend on their life stage and nutritional state. Vegetative cells of phytoplankton occupy a broad range of densities from 1030 to 1200 kg/$m^{3}$ [10], with most species that are not heavily silicified or calcareous having densities near 1050 kg/$m^{3}$ [11]. Run 4 used the same particle diameter as Run 3 but with a particle density of $\rho_{p}$ = 950 kg/$m^{3}$ and Run 5 used the same particle diameter as Run 3 but with a particle density of $\rho_{p}$ = 1050 kg/$m^{3}$. Comparisons of Runs 3–5 highlight the impact of particle density on the residence time and rate of strain.

The numerical model requires the specification of the pressure as a boundary condition at the inlet and outlet of the modeling domain. These boundary conditions can be interpreted as a specification of the bathyphotometer deployment depth, and varying these boundary conditions will highlight the sensitivity of the model results to the depth of the SIM-BATH in the field. Runs 1–5 were conducted with a pressure of *p* = 101.3 kPa, which corresponds to deployment at sea level. Run 6 used the same particle diameter and density as in Run 1, but the pressure at the inlet and outlet was set to *p* = 199.1 kPa, corresponding to a depth of deployment of 10 m based on the hydrostatic assumption. Run 7 was set up in the same way as Run 1 and Run 6, but the inlet and outlet pressures were defined as *p* = 1081.3 kPa, corresponding to a depth of deployment of 100 m.

## 3. Results

### 3.1. Residence Time Analysis

**Figure 3:**
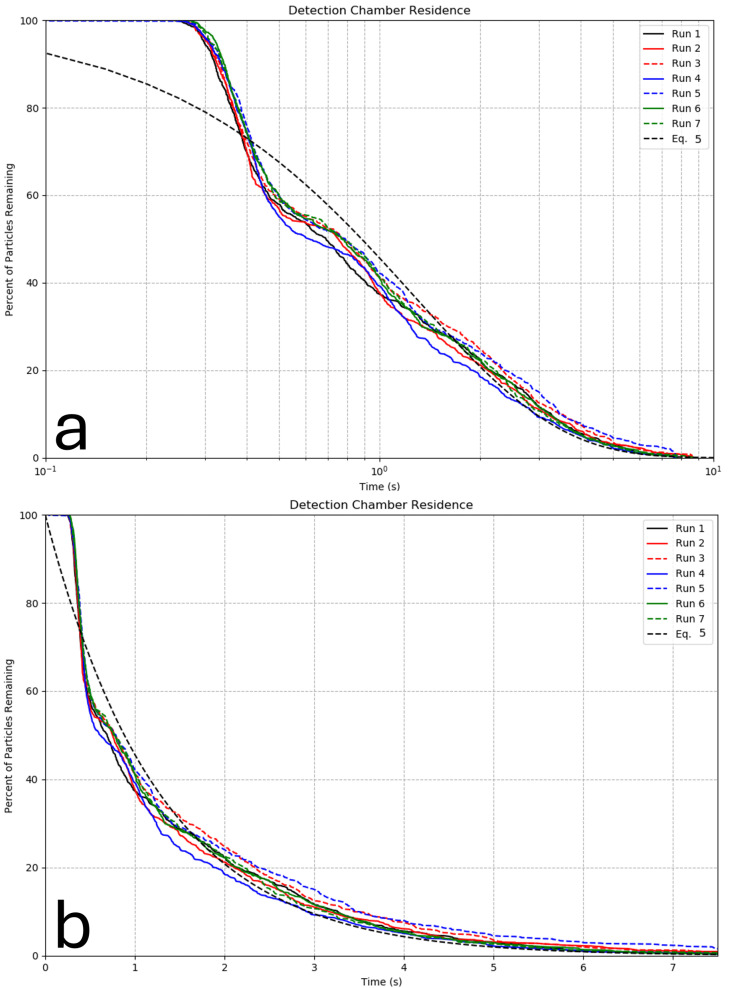
Distribution of particles remaining in the detection chamber over time for the runs in Table 1 and from Equation (5): The x-axis represents the time particles have spent in the detection chamber (**a**) on a logarithmic scale and (**b**) on a linear scale. The y-axis represents the percent of particles remaining in the detection chamber.

To quantify the residence time of particles in the detection chamber, the percentage of particles remaining in the detection chamber as a function of time was calculated for the seven model runs listed in Table 2 and shown in Figure 3. The figure also shows the percentage of particles remaining in the detection chamber using the analytical function in Equation (5). The percentage of particles remaining for specific times corresponding to the flash durations of certain species is also shown in Table 2.

As shown in Figure 3 and Table 2, all particles remain in the detection chamber until at least *t* = 0.25 s in all seven runs. After that time, the number of particles remaining in the detection chamber decays exponentially. All particles leave the detection chamber by about *t* = 8–10 s. There is very low sensitivity of particle residence time to the variations in sizes, density of particles, or the depth of the instrument deployment.

Bioluminescent flash durations for most autotrophic and heterotrophic dinoflagellates range from around 0.1 s to 0.25 s, and 0.2 s is the average flash duration for the heterotrophic dinoflagellate *Gonyaulax polyedra* [8,12,13]. This suggests that, in general, the total first flash of most dinoflagellates will be measured by the bathyphotometer. As shown in Figure 3 and Table 2, around 60% of particles remain in the detection chamber at *t* = 0.5 s, which suggests that approximately 60% of dinoflagellates remain in the detection chamber long enough to produce a secondary flash, which can occur if the rate of strain experienced by the organism again exceeds the threshold rate of strain. As a result, the measured BL potential might include multiple flashes from about 60% of candidate dinoflagellates. At the same time, around 0.5 s represents an average flash duration for the heterotrophic dinoflagellates *Noctiluca scintillans* [14], as well as the flash duration of the copepod *Metridia longa* [15,16]. Therefore, the bathyphotometer will measure the Total Mechanically Stimulated Light from around 60% of those plankton. At *t* = 1 s, only 40% of particles remain in the detection chamber, which means that around 40% of *Noctiluca scintillans* and *Metridia longa* will be able to flash twice depending on the possibility of re-stimulation in the detection chamber. For the ctenophore *Beroe cucucmis*, which has a flash duration reaching 1.4 s [16] or even 2.2 s [15], the TMSL of only around 20–25% of organisms will be presented in the BL potential measured by the bathyphotometer. For the other 75–80% of *Beroe*, only part of their flashes will be measured by the SIM-BATH.

The residence time curve corresponding to Equation (5) shows a lower percentage of particles remaining compared to the CFD results until around *t* = 0.4 s, which is likely due to the assumption of well-mixed particles used in the Herren report. The curves for the seven CFD runs and the curve for Equation (5) are in very good agreement after 1.3 s, which corresponds to the time scale in Equation (5): ${\left(-\frac{t}{\rho V}\frac{dm}{dt}\right)}^{-1}$= 1.28 s. After that time, the particles recirculate and mix more thoroughly, more closely following the well-mixed assumption.

### 3.2. Rate of Strain Analysis

**Figure 4:**
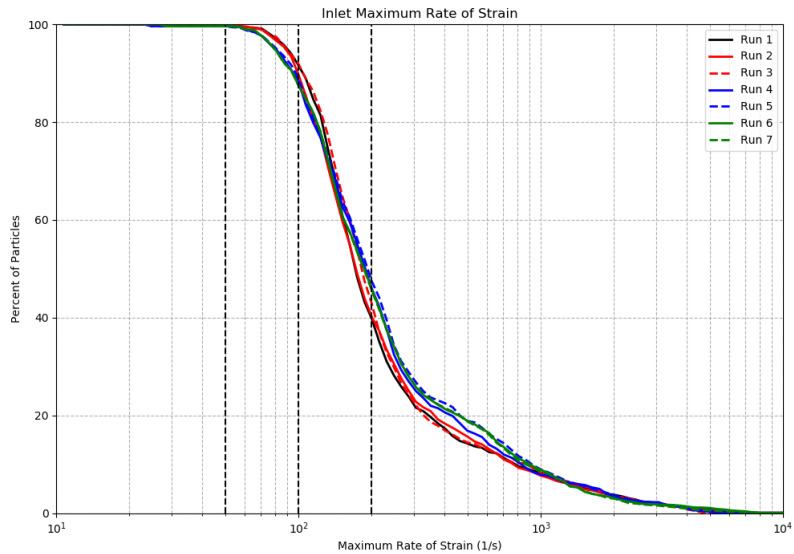
Percent of particles stimulated in the inlet as a function of threshold rate of strain. Three vertical dashed black lines correspond to threshold rates of strain of 50, 100, and 200 $s^{-1}$.

**Figure 5:**
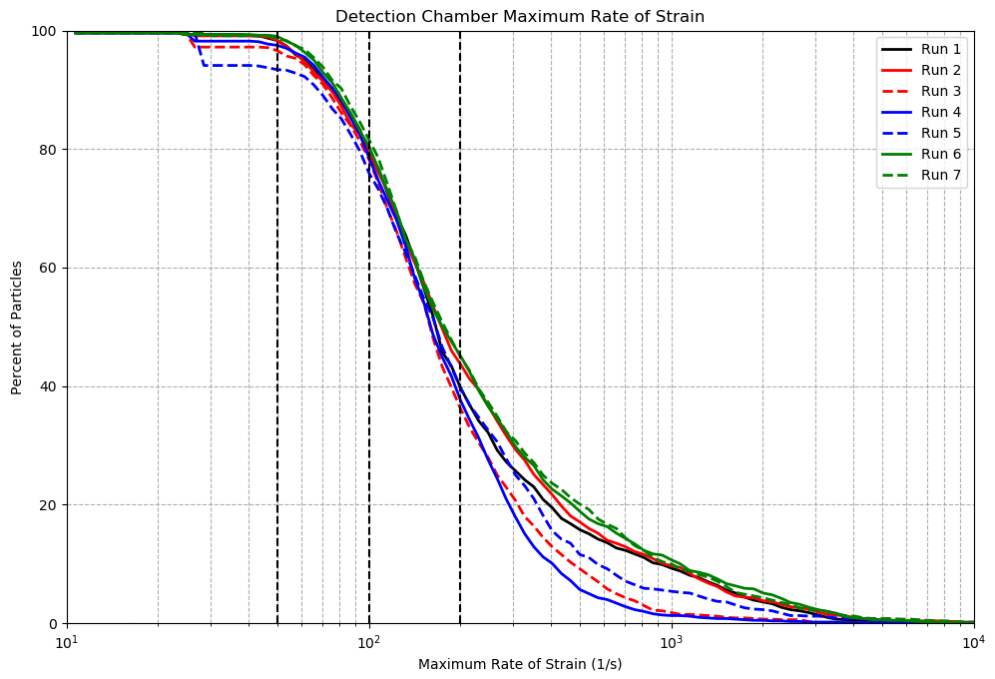
Percent of particles stimulated in the detection chamber as a function of the threshold rate of strain. Three vertical dashed black lines correspond to threshold rates of strain of 50, 100, and 200 $s^{-1}$.

**Figure 6:**
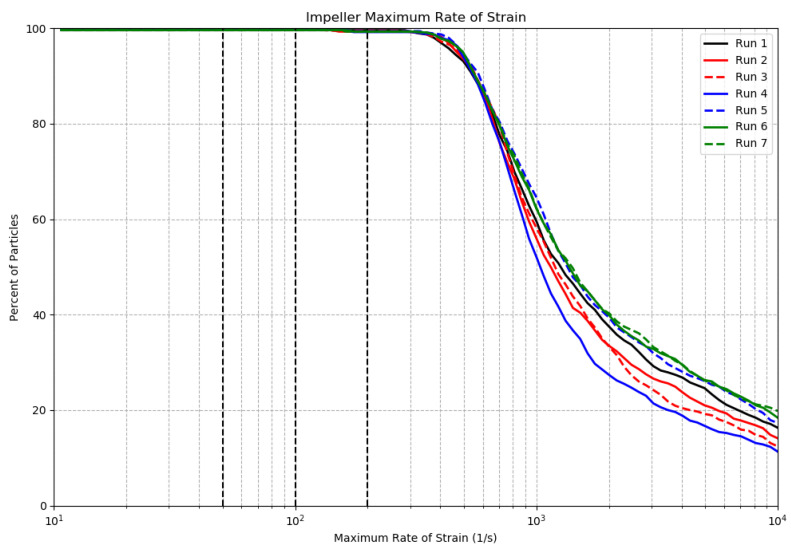
Percent of particles stimulated in the pump impeller region as a function of the threshold rate of strain. Three vertical dashed black lines correspond to threshold rates of strain of 50, 100, and 200 $s^{-1}$.

Numerous studies have been conducted to determine the rate of strain required to stimulate bioluminescence in different taxa [12,17,18]. These studies found that the rate of strain required for stimulation in steady laminar flow varies between 20 and 300 $s^{-1}$. For the runs in Table 1, Figure 4 plots the percent of particles stimulated in the S-shaped inlet as a function of the threshold rate of strain to cause that stimulation. Figure 5 and Figure 6 present similar estimations for the detection chamber and the pump impeller, respectively. Table 3 presents the percent of particles experiencing rates of strain exceeding 50, 100, and 200 $s^{-1}$ in the inlet and in the detection chamber.

Comparisons of Figure 4 and Figure 5 show that a higher percentage of particles experienced a given rate of strain in the inlet compared to the detection chamber for all considered runs. This is also supported by Table 3, where 100% of particles experienced a rate of strain exceeding 50 $s^{-1}$ in the inlet area. In the detection chamber, this percentage was approximately 93%. On average, approximately 90% of particles in the inlet experienced a rate of strain exceeding 100 $s^{-1}$, compared to about 80% of particles in the detection chamber. The S-shaped inlet was designed with the objective of minimizing marine organisms’ exposure to high rates of stain in order to avoid pre-stimulation prior reaching the impeller. The rate of strain of 100 $s^{-1}$ corresponds to a pressure of 1 dyn/${\mathrm{cm}}^{2}$ at sea level dynamic viscosity, and it is a well-known threshold for the stimulation of the dinoflagellate *Gonyaulax polyedra* [17]. Our results demonstrate that 90% of the dinoflagellate *Gonyaulax polyedra* will receive sufficient mechanical stimulation in the inlet to initiate mechanically stimulated bioluminescence prior to passing the impeller, and as a result some portion of their flash will not be recorded inside of the detection chamber. At the same time, based on the results of the previous section, around 60% of autotrophic dinoflagellates remain in the detection chamber for at least 0.5 s, and 80% of them experience a rate of strain above the threshold of 100 $s^{-1}$ (Table 3). As shown in Figure 6, 100% of particles experienced a rate of stain exceeding 300 $s^{-1}$ in the pump impeller region, which is the maximum rate of strain considered in the text for the mechanical stimulation of bioluminescence.

For low rates of strain (below 50 $s^{-1}$), Run 3 and Run 5 deviated from the baseline run. Analysis of particle paths (not shown here), demonstrated that this discrepancy can be attributed to particles that settle in the detection chamber, with the percent of particles settling proportional to the mass of the particles. Rate of strain plots in all regions show that some particles experience very high rates of strain, in some cases well over 1000 $s^{-1}$. This can be attributed to the interaction of a small percentage of the particles with the boundary layer in each region, where strain is very high compared to the surrounding flow due to the considerable velocity gradient near the wall.

## 4. Discussion

We developed a numerical model of a pump-through bathyphotometer. The dimensions of the UBAT instrument were used to create the domain for the numerical model, called the SIM-BATH. The SIM-BATH has all elements of a pump-through bathyphotometer, including an S-shaped inlet, two pumps for mechanical stimulation and flow rate control, a detection chamber, and an outlet. We conducted CFD simulations of flow through the SIM-BATH, using Lagrangian particles as an approximation of marine taxa. From these simulations, we presented a distribution of the residence times of particles in the detection chamber of the SIM-BATH, as well as a statistical analysis of the rate of strain experienced by particles passing through the inlet and the detection chamber. Our modeling results demonstrate a very low sensitivity of particle residence time and rate of strain in the detection chamber to the variations in their sizes, density, or the depth of the instrument deployment.

We found that all particles remain in the detection chamber for at least 0.25 s. This suggests that most autotrophic and heterotrophic dinoflagellates, including *C. horrida*, *G. polyedra*, *L. polyedra*, *P. fusiformis*, *P. lunula*, and *T. fusus*, will have their total first flash measured by the bathyphotometer because their commonly accepted flash durations are less than 0.25 s [19]. One notable exception is the large heterotrophic dinoflagellate *P. noctiluca*, which has a flash duration of about 0.5 s. Only about 60% of *P. noctiluca* passing through the detection chamber will remain inside long enough for their total first flash to be recorded. Concerning other bioluminescent taxa, our results demonstrate that the bathyphotometer will measure the total first flash from around 60% of the copepod *M. longa*, based on their flash duration from the literature [19]. Our simulations have also shown that for the ctenophore *B. cucumis*, the total first flash will be recorded for only around 25% of organisms, and for the remaining 75%, only part of their flashes will be measured inside of the detection chamber.

We also found that the rate of strain within the S-shaped inlet is sufficient to produce pre-stimulation of many dinoflagellates. While passing through the inlet, 90% of particles experience a rate of strain exceeding 100 $s^{-1}$. *C. horrida*, *G. polyedra*, *P. fusiformis*, and *T. fusus* are highly likely to experience pre-stimulation as their commonly accepted threshold rates of strain are below this value [19]. *P. lunula* and *L. polyedra* have threshold rates of strain of 200 $s^{-1}$ and 320 $s^{-1}$, respectively [19]. As a result, about 40% of *P. lunula* and 25% of *L. polyedra* may experience pre-stimulation. The copepod *M. longa*, with a threshold rate of strain of 510 $s^{-1}$, has a very low likelihood of pre-stimulation [19].

Finally, we find that the long residence time of many particles coupled with the high rate of strain in some areas of the detection chamber may produce re-stimulation of certain taxa as they continue to circulate. For dinoflagellates with a short flash duration and low rate of strain threshold like *C. horrida*, *G. polyedra*, *P. fusiformis*, and *T. fusus*, 50% or more may undergo at least one additional stimulation while in the detection chamber [19].

Our results lend themselves to a discussion of some issues with the UBAT. First, we observe high rates of strain in the instrument prior to the detection chamber. While the inlet is likely effective as a light baffle, the two elbows in the S-shaped inlet create pockets for recirculation, and the narrow inlet diameter produces a high-shear boundary layer that extends well into the interior of the pipe. In addition, we observe that the detection chamber does not produce consistent residence times. Half of the particles are quickly directed through the outlet in under a second, while the rest remain within the chamber for as long as ten seconds. From this, some organisms’ first flashes are not fully recorded, while others may be stimulated to exhaustion as they recirculate. Consistent BL potential data collection could benefit from more uniform residence times for all organisms. Finally, the distance particles must travel after being stimulated by the pump impeller but prior to the start of the detection chamber may result in some light emission from the stimulation not being recorded.

## Figures and Tables

**Table 1:** List of the model runs.

Run	Diameter	Density	Pressure
1	2 μm	1000 kg/$m^{3}$	101.3 kPa
2	20 μm	1000 kg/$m^{3}$	101.3 kPa
3	200 μm	1000 kg/$m^{3}$	101.3 kPa
4	200 μm	950 kg/$m^{3}$	101.3 kPa
5	200 μm	1050 kg/$m^{3}$	101.3 kPa
6	2 μm	1000 kg/$m^{3}$	199.3 kPa
7	2 μm	1000 kg/$m^{3}$	1081.3 kPa

**Table 2:** List of the model runs with the percent of particles remaining in the detection chamber at the durations of interest.

Run	0.25 s	0.5 s	1.0 s	1.4 s	2.2 s
1	100%	58.3%	37.9%	30.9%	20.1%
2	100%	57.4%	38.4%	30.0%	19.9%
3	100%	60.4%	43.1%	35.1%	24.2%
4	100%	56.1%	40.4%	27.8%	18.0%
5	100%	62.3%	45.6%	34.6%	26.6%
6	100%	60.6%	41.6%	30.0%	19.9%
7	100%	59.2%	41.8%	31.5%	21.0%

**Table 3:** Percent of particles experiencing a threshold rate of strain of 50, 100, and 200 $s^{-1}$ in the inlet and detection chamber.

Run	Inlet			Detection Chamber		
	**50** $s^{-1}$	**100** $s^{-1}$	**200** $s^{-1}$	**50** $s^{-1}$	**100** $s^{-1}$	**200** $s^{-1}$
1	100%	91.6%	39.7%	98.3%	79.3%	39.7%
2	100%	89.7%	40.1%	98.3%	79.3%	43.7%
3	100%	91.9%	42.8%	96.6%	78.3%	36.2%
4	100%	88.3%	45.9%	97.5%	78.5%	37.6%
5	100%	89.0%	47.4%	93.4%	76.0%	39.7%
6	100%	87.5%	45.8%	99.0%	80.5%	45.1%
7	100%	87.5%	45.5%	98.6%	81.6%	44.9%

**Figure A1:**
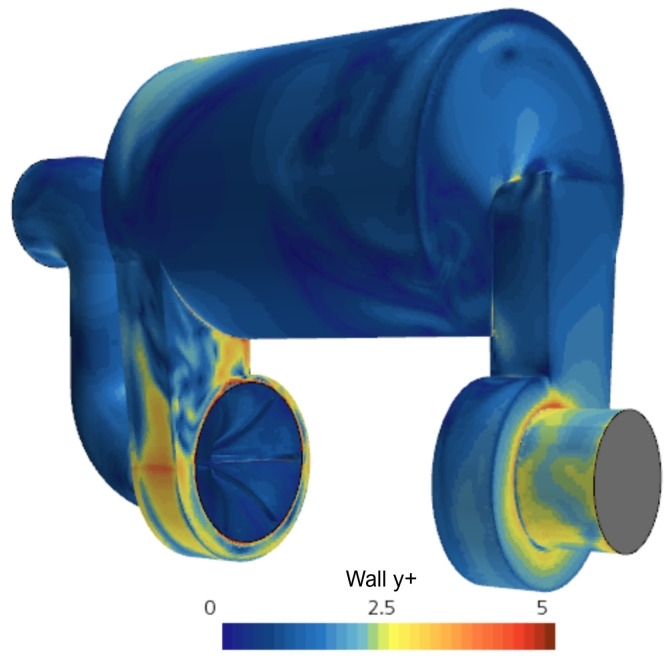
Wall y+ throughout the fluid domain as calculated from Equation (A6).

**Figure A2:**
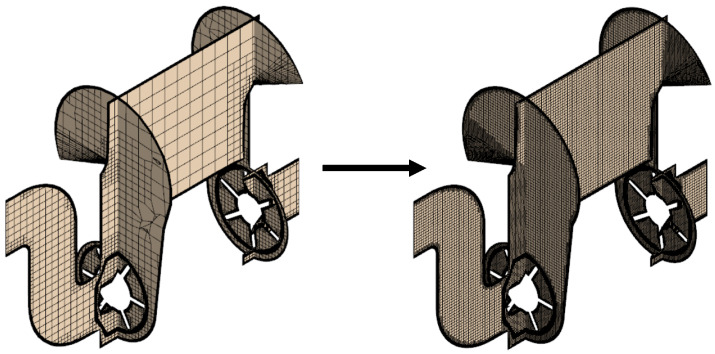
Mesh refinement from 0.25 million cells to 2.7 million cells. Visible are the orthogonal hexahedral volume cells, as well as the high aspect ratio boundary layer cells at the walls.

## Data Availability

The raw output files for the model runs analyzed in this paper, as well as data sufficient to regenerate the figures, tables, and other results in this paper, are stored on US Naval Research Laboratory computers and will be made available to members of the scientific community upon request. To obtain the data, please contact the corresponding author.

## Abbreviations

The following abbreviations are used in this manuscript:

BL	bioluminescence
UBAT	Underwater Bioluminescence Assessment Tool
TMSL	Total Mechanically Stimulated Light
CFD	Computational Fluid Dynamics
SIM-BATH	Simulated Bathyphotometer
CAD	Computer-Assisted Design
FVM	finite volume method
RANS	Reynolds-Averaged Navier–Stokes
UCSB	University of California, Santa-Barbara

## Appendix A

### Appendix A.1. Turbulence Model

The low-Reynolds *k*-ω SST model was selected to model the flow [9]. The model uses two transport variables: *k*, turbulent kinetic energy, and ω, specific dissipation rate. These parameters are used to determine turbulent eddy viscosity and close the RANS equations. To alleviate sensitivity to free-stream ω, the SST model applies a blending function outside the boundary layer to convert ω into ϵ. The *k*-ω SST solution for turbulent eddy viscosity is provided in Equations (A1)–(A4):

(A1) $$\mu_{t}=\rho kT$$

(A2) $$T=min\left(\frac{a^{*}}{\omega },\frac{a_{1}}{SF_{2}}\right)$$

(A3) $$S=\left|\frac{1}{2}\left(\nabla \vec{v}+\nabla {\vec{v}}^{T}\right)\right|$$

(A4) $$F_{2}=tanh\left[max{\left(\frac{2\sqrt{k}}{\beta^{*}\omega d},\frac{500\nu }{d^{2}\omega }\right)}_{2}\right]$$

Here, *T* is the turbulent time scale; $a^{*}$, $a_{1}$, and $\beta^{*}$ are the model coefficients presented in Table A1 [20]; *S* is the modulus of the mean strain rate tensor; and $F_{2}$ is the blending function, where *d* is the wall distance.

**Table A1:** k−ω model coefficients used in the SIM-BATH simulation.

Coefficient	Value
$a^{*}$	1.0
$a_{1}$	0.31
$\beta^{*}$	0.09

### Appendix A.2. Grid Design

Because the *k*-ω SST model can model the entirety of the boundary layer, the boundary layer mesh must extend into the viscous sublayer. The boundary layer thickness was sized using the turbulent boundary layer thickness equation for each region (Equation (A5)), where *x* is the approximate pipe length and $Re_{x}$ is the local Reynolds number. The local wall y+, found using Equation (A6), is plotted in Figure A1, where *y* is half the first cell thickness, ν is the kinematic viscosity, and $u^{*}$ is the local friction velocity. To model the viscous sublayer, the y+ should be less than five and ideally near one [21]. The value of y+ ranges from approximately 0.2 to 1.0 across the majority of the domain, with local patches up to 3.0 near the impellers while still in the SIM-BATH domain where the cell thickness is larger. The boundary layer is modeled using 15 prism layer cells extending to 20% of the anticipated boundary layer thickness. When sizing the mesh, the characteristic velocity for stationary components is the inlet velocity for a volumetric flow rate of 0.33 L/s. The characteristic length is the diameter of the S-shaped inlet. Impeller characteristic velocities are their respective tip speeds, with the characteristic length being the diameter of the impeller. The resulting mesh parameters are provided in Table A2.

(A5) $$\delta =0.37\times Re_{x}^{-0.2}$$

(A6) $$y=\frac{y_{+}\nu }{u^{*}}$$

**Table A2:** Comparison of boundary layer sizing for each SIM-BATH region.

Region	Characteristic Velocity	Characteristic Length	First Cell Thickness	Boundary Layer Thickness
Stationary	0.415 m/s	3.18 cm	42.0 μm	0.70 cm
Pump Impeller	2.525 m/s	4.02 cm	8.8 μm	0.59 cm
Flow Impeller	1.262 m/s	4.02 cm	15.0 μm	0.68 cm

### Appendix A.3. Grid Convergence

A grid convergence study was performed to determine a grid-independent solution for Run 1. A set of grids increasing in resolution was created. The generated meshes for the least and most refined models are shown in Figure A2.

The optimal grid size was selected by recording the change in volumetric flow rate from the previous grid to the next. We defined the optimal grid as the smallest grid such that there is less than a 1% change in the flow rate from the previous grid. Between 1.6 million and 2.7 million cells, the change in average flow rate is 0.003 L/s, less than 1% of the total flow rate and well within the standard deviation of 0.009 L/s. This standard deviation represents the temporal variability of the volumetric flow rate due to the unsteady pumping effect of the impellers. As a result, the final grid size used to produce the following results has a cell count of 2.7 million with a flow rate of $\frac{1}{V}\frac{dm}{dt}$ = 0.345 L/s. To establish an appropriate time step for the simulation, we referred to the rotation rates of the impellers. For impeller rotation, one to five degrees of rotation per time step is generally sufficient [22]. In our model, we used a time step size of Δt = 0.25 ms, where each time step corresponds to approximately two degrees of rotation on the pump impeller and one degree of rotation on the flow impeller.

**Table A3:** Convergence of the time-averaged volumetric flow rate from 0.5 s to 1.0 s of model time.

Cell Count	Volumetric Flow Rate (L/s)
$2.5\times 10^{5}$	0.286 ± 0.006
$4.8\times 10^{5}$	0.319 ± 0.004
$6.6\times 10^{5}$	0.328 ± 0.005
$1.6\times 10^{6}$	0.342 ± 0.008
$2.7\times 10^{6}$	0.345 ± 0.009

### Appendix A.4. Residuals

In STAR CCM+, residuals are calculated at a given iteration using the following normalized quantity *R* and its constituents, as shown in Equations (A7)–(A9).

(A7) $$R=\frac{R_{RMS}}{R_{NORM}}$$

(A8) $$R_{RMS}=\sqrt{\frac{1}{n}\sum_{N}r^{2}}$$

(A9) $$R_{NORM}=max\left(R_{1},R_{2},...,R_{5}\right)$$

where *r* is the absolute error of the transport quantity in a given cell and $R_{1}$–$R_{5}$ are the root mean square (RMS) residuals of the first five iterations. $R_{NORM}$ is recalculated at every time step after five internal iterations. Time-step residuals for each variable are provided in Table A4.

**Table A4:** Residuals for each variable in the SIM-BATH model at a time of 1.0 s.

Variable	*R*	$R_{\mathrm{NORM}}$	$R_{\mathrm{RMS}}$
Continuity	9.02 × $10^{-4}$	3.304	2.98 × $10^{-3}$
X-Momentum	7.35 × $10^{-4}$	9.967	7.33 × $10^{-3}$
Y-Momentum	8.41 × $10^{-4}$	9.831	8.27 × $10^{-3}$
Z-Momentum	1.41 × $10^{-2}$	3.533	4.98 × $10^{-2}$
ω	5.26 × $10^{-5}$	54.481	2.87 × $10^{-3}$
*k*	2.39 × $10^{-3}$	7.004	1.67 × $10^{-2}$

### Appendix A.5. Lagrangian Particles

The equation of motion [23] is given in Equation (3), where $\vec{F_{D}}$ and $\vec{F_{P}}$ are surface force vectors corresponding to the effects of drag and pressure, $\vec{F_{G}}$ is a body force vector representing the force of gravity, $m_{p}$ is the mass of the particle, and $\frac{d\vec{v_{p}}}{dt}$ is the time rate of change of the particle’s velocity vector. $\vec{F_{D}}$ is calculated in Equation (A10) using the drag coefficient $C_{d}$, where $\left(\vec{v}-\vec{v_{p}}\right)$ is the relative velocity vector of the particle with respect to the local velocity vector of the fluid, ρ is the local fluid density, and $A_{p}$ is the surface area of the particle.

(A10) $$\vec{F_{D}}=\frac{1}{2}C_{d}\rho A_{p}\left|\vec{v}-\vec{v_{p}}\right|\left(\vec{v}-\vec{v_{p}}\right)$$

The Schiller–Naumann correlation provides the value for $C_{d}$ as follows in Equation (A11), where $Re_{p}$ is the particle Reynolds number.

(A11) $$C_{d}=\left\{\begin{array}{cc} \frac{24}{Re_{p}}\left(1+0.15Re_{p}^{0.687}\right) & Re_{p}<1000\\ 0.44 & Re_{p}>1000 \end{array}\right\}$$

The particle Reynolds number $Re_{p}$ [24] can be calculated as in Equation (A12), where $D_{p}$ is defined as the characteristic length of the particle, in this case the particle diameter, and μ is the local dynamic viscosity of the fluid.

(A12) $$Re_{p}=\frac{\rho \left|\vec{v}-\vec{v_{p}}\right|D_{p}}{\mu }$$

$F_{P}$ is calculated as in Equation (A13), where $V_{p}$ is the particle volume and ∇p is the local pressure gradient in the fluid. Because of the low particle volume and pressure gradient, it is not as large a contributor as the drag force.

(A13) $$\vec{F_{P}}=-V_{p}\nabla p$$

The body force $F_{G}$ is the product of the particle mass $m_{p}$ and the acceleration due to gravity. The gravity vector $\vec{g}$ is pointed downwards, as shown in Equation (A14).

(A14) $$\vec{F_{G}}=m_{p}\vec{g}$$
